# Effects of Three Different Types of Antifreeze Proteins on Mouse Ovarian Tissue Cryopreservation and Transplantation

**DOI:** 10.1371/journal.pone.0126252

**Published:** 2015-05-04

**Authors:** Jaewang Lee, Seul Ki Kim, Hye Won Youm, Hak Jun Kim, Jung Ryeol Lee, Chang Suk Suh, Seok Hyun Kim

**Affiliations:** 1 Department of Obstetrics and Gynecology, Seoul National University Bundang Hospital, Seongnam, Gyeonggi-do, Korea; 2 Department of Obstetrics and Gynecology, Seoul National University College of Medicine, Seoul, Korea; 3 Division of Polar Life Sciences, Korea Polar Research Institute, Incheon, Korea; 4 Department of Chemistry, Pukyong National University, Busan, Korea; University Hospital of Münster, GERMANY

## Abstract

**Background:**

Ovarian tissue (OT) cryopreservation is effective in preserving fertility in cancer patients who have concerns about fertility loss due to cancer treatment. However, the damage incurred at different steps during the cryopreservation procedure may cause follicular depletion; hence, preventing chilling injury would help maintain ovarian function.

**Objective:**

This study was designed to investigate the beneficial effects of different antifreeze proteins (AFPs) on mouse ovarian tissue cryopreservation and transplantation.

**Methodology:**

Ovaries were obtained from 5-week-old B6D2F1 mice, and each ovary was cryopreserved using two-step vitrification and four-step warming procedures. In Experiment I, ovaries were randomly allocated into fresh, vitrification control, and nine experimental groups according to the AFP type (FfIBP, LeIBP, type III) and concentration (0.1, 1, 10 mg/mL) used. After vitrification and warming, 5,790 ovarian follicles were evaluated using histology and TUNEL assays, and immunofluorescence for τH2AX and Rad51 was used to detect DNA double-strand breaks (DSBs) and repair (DDR), respectively. In Experiment II, 20 mice were randomly divided into two groups: one where the vitrification and warming media were supplemented with 10 mg/mL LeIBP, and the other where media alone were used (control). Ovaries were then autotransplanted under both kidney capsules 7 days after vitrification together with the addition of 10 mg/mL LeIBP in the vitrification-warming media. After transplantation, the ovarian follicles, the percentage of apoptotic follicles, the extent of the CD31-positive area, and the serum FSH levels of the transplanted groups were compared.

**Principal Findings:**

In Experiment I, the percentage of total grade 1 follicles was significantly higher in the 10 mg/mL LeIBP group than in the vitrification control, while all AFP-treated groups had significantly improved grade 1 primordial follicle numbers compared with those of the vitrification control. The number of apoptotic (TUNEL-positive) follicles was significantly decreased in the groups treated with 1 and 10 mg/mL LeIBP. The proportion of τH2AX-positive follicles was significantly reduced in all AFP-treated groups, while the proportion of Rad51-positive follicles was significantly decreased in only the FfIBP- and LeIBP-treated groups. In Experiment II, after autotransplantation of OT vitrified with 10 mg/mL of LeIBP, the percentage of total grade 1 and primordial grade 1 follicles, and the extent of the CD31-positive area, were increased significantly. Moreover, the levels of serum FSH and the percentage of TUNEL-positive follicles were significantly lower in the LeIBP-treated than in the control group.

**Conclusion:**

A supplementation with high concentrations of AFPs had protective effects on follicle preservation during OT vitrification-warming procedures. The group treated with LeIBP was protected most effectively. The beneficial effects of LeIBP were also observed after autotransplantation of vitrified-warmed OT. Further studies are necessary to determine the exact mechanism of these protective effects.

## Introduction

Ovarian tissue (OT) cryopreservation is an effective option for preserving fertility in cancer patients who have concerns about fertility loss due to cancer treatment. Currently, OT banking is the only applicable method for prepubertal cancer patients who cannot undergo controlled ovarian hyperstimulation, for those who face a delay in chemotherapy, or for those who do not use embryo banking. Although fertility preservation using OT has aforementioned advantages, it is still experimental and some problems remain to be solved, such as cryodamage, ischemic damage and re-implantation of malignant cells [1].

Because the damage occurring during the cryopreservation procedure may cause follicular depletion, prevention of chilling injury is the most important requirement for maintaining ovarian function. Recent research has focused on developing methods to prevent follicle depletion and improve ovarian function after ovarian tissue cryopreservation. These methods include using computerized freezing and vitrification procedures, various slow freezing protocols and vitrification procedures [2], genetic manipulation [3], different cryodevices [4], different transport times and temperatures [5], several different cryoprotective agents [6], and other approaches [7]. Despite these efforts, cryodamage still occurs, resulting in the impairment of ovarian function. Therefore, we attempted to reduce the cryodamage through lowering the freezing point and prevent ice-recrystallization during vitrification and warming procedure.

Antifreeze proteins (AFPs) lower the freezing point of a solution in a non-colligative manner, leading to an increase in the difference between the melting point and the freezing point. This phenomenon is known as thermal hysteresis (TH) and involves binding of AFPs to the surfaces of ice crystals [8]. In 1969, DeVries and his colleague isolated the first AFP from Antarctic fish [9]. Since then, AFPs (or ice-binding proteins [IBPs]) that permit survival in subzero environments have also been reported in vertebrates, insects, plants, fungi, and bacteria [10]. Moreover, AFPs inhibit ice recrystallization (IR), thus protecting cellular membranes in polar organisms [11]. IR refers to the growth of larger ice grains at the expense of smaller ones, a phenomenon that is fatal to cells and leads to cold damage and cell death [12, 13]. Except for fish, most psychrophilic organisms inhibit IR to protect their cell membranes from cryodamage in order to survive extremely icy conditions.

Many different types of AFPs, with different amino acid sequences, molecular weights, ice-binding affinities, TH activities, origins, and structural differences have been identified. AFPs are classified as hyperactive or moderately active according to their TH activity levels. Most fish AFPs exhibit moderate TH activity at about 1°C [14], while hyperactive AFPs, found in many insects, plants, and bacteria, exhibit TH activities at more than 1°C [15, 16].

In the current study, three different AFPs were examined (Table 1): one hyperactive AFP from *Flavobacterium frigoris* (FfIBP), a moderately active IBP from an arctic yeast (*Leucosporidium* sp.; currently known as *Glaciozyma*), and a fish type III AFP. FfIBP is an AFP subtype extracted from the gram-negative bacterium *F*. *frigoris* PS1, which is found in Antarctic sea ice [17] and has a TH activity of approximately 2.5 K at 50 μM [18]. The arctic yeast *Leucosporidium* sp. produces a glycosylated ice-binding protein (LeIBP) with a molecular mass of 25 kDa and can lower the freezing point of water 0.4 K below its melting point [19]. Type III AFP has a TH activity of approximately 0.9 K and is only 66 amino acid residues long. Although these IBPs have different TH activities, and they all inhibit IR, which cannot be quantitated. A variety of studies have reported diverse effects by AFPs on the hypothermal preservation of an insulinoma cell line [20], *Escherichia coli* [21], red blood cells [11], bovine sperm [22], and mouse oocytes [23]. Through these previous studies, we could assume that AFPs also have preventive effects of cryodamage even in the cryopreservation of OT. Thus, in the current study, we tried to evaluate the cryoprotective effects of three different types of AFPs on mouse ovaries during cryopreservation procedures. The results demonstrated that these effects were also beneficial for OT transplantation. Based on these results, we suggest that addition of AFPs on vitrification-warming media is useful for the preservation of OT in female cancer patients.

**Table 1:** Characteristic of AFPs used in this study.

	*Type III AFP*	*LeIBP*	*FfIBP*
**Mass (kDa)**	6.5	~ 27	~ 25.3
**Structure**	globular	β-helix	β-helix
**Natural source**	Ocean pout, Wolfish and Eelpout	*Glaciozyma* sp.	*Flavobacterium frigoris*
**Thermal Hysteresis (TH)**	~ 1.5°C at 3 mM	0.42°C at 0.4 mM	2.5°C at 50 μM
**Reference**	Structure-function relationship in the globular type III AFP: identification of a cluster of surface residues required for binding to ice	Characterization of the ice-binding protein from Arctic yeast Leucosporidium sp. AY30	Structure-based characterization and antifreeze properties of a hyperactive ice-binding protein from the Antarctic bacterium Flavobacterium frigoris PS1

## Materials and Methods

### Experimental animals and ethics

Five-week-old B6D2F1 female mice (Orient Co., Seongnam, South Korea) were housed under a 12-h light/dark cycle at 22°C and provided food ad libitum. The experimental protocols and animal handling procedures were performed with the approval of the Institutional Animal Care and Use Committee of Seoul National University Bundang Hospital (BA1304-126-029-01).

### Vitrification and warming of whole mouse ovaries (Experiment I)

**Fig 1:**
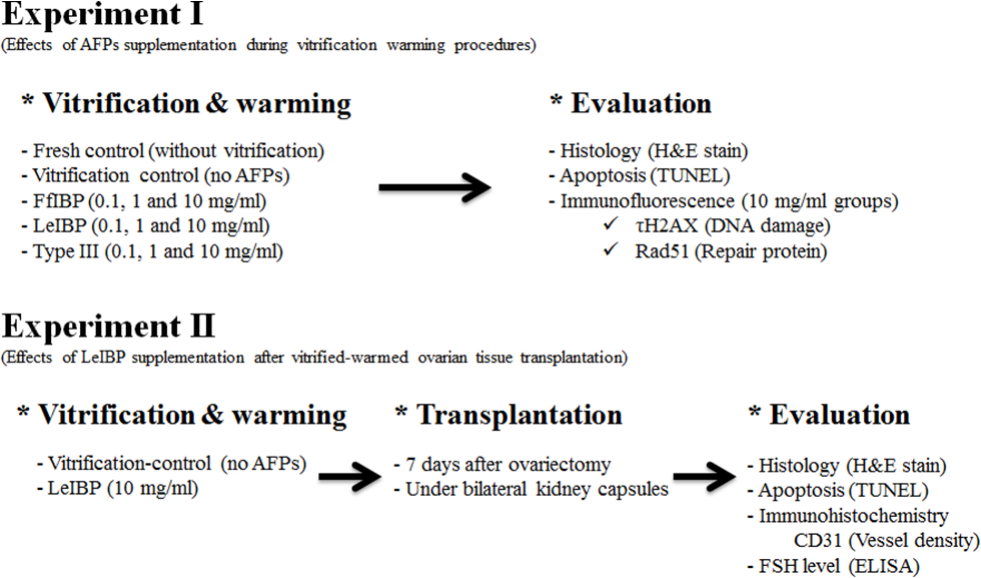
A schematic figure showing two experimental schemes. This experimental flow was constructed to evaluate the effects of three different antifreeze proteins.

A schematic diagram showing the design for Experiment I (Fig 1). Whole ovaries were obtained from mice after cervical dislocation and were randomly assigned to one of three groups: fresh control, vitrification control, or AFP-treated. The AFP-treated group was further divided into nine subgroups according to AFP type (e.g., FfIBP, LeIBP, and type III AFP) and concentration (0.1, 1, and 10 mg/mL). A total of 240 whole ovaries were obtained from 120 mice. A pilot study showed that type III AFPs could have beneficial effects on OT vitrification and warming when used at 5 mg/mL [24].

Ovaries were vitrified using a two-step process [6]. First, they were equilibrated for 10 min at room temperature in Dulbecco’s phosphate-buffered saline (D-PBS) supplemented with 20% (v/v) fetal bovine serum (FBS; Gibco, Carlsbad, CA, USA), 7.5% (v:v) ethylene glycol (EG; Sigma-Aldrich, St. Louis, MO, USA), and 7.5% (v/v) dimethyl sulfoxide (Sigma-Aldrich). Ovaries were then placed into the vitrification medium (D-PBS containing 20% FBS, 20% EG, 20% dimethyl sulfoxide, and 0.5 M sucrose (Sigma-Aldrich)) for 5 min at room temperature. To enhance heat conductivity and eliminate any remaining water, each ovary was then put on an electron microscopic copper grid (JEOL, Tokyo, Japan) and plunged directly into liquid nitrogen. The vitrified ovaries were then placed into 1.5-mL cryovials (Nunc, Roskilde, Denmark) filled with liquid nitrogen.

At least 1 week after vitrification, ovaries were warmed as follows. First, the ovaries were exposed to air for 10 sec. They were then rehydrated at room temperature via sequential 5-min equilibrations in 1, 0.5, 0.25, and 0 M sucrose solutions. D-PBS supplemented with 20% FBS was used as the basal medium for both vitrification and warming. For the AFP-treated groups, the medium was supplemented with AFP during both vitrification and warming.

### Autotransplantation of cryopreserved ovaries (Experiment II)

Experiment II was performed to determine whether the cryoprotective effects of LeIBP could be also seen in OT after transplantation (Fig 1). In total, 20 B6D2F1 mice were randomly divided into two groups: one group received ovaries treated with 10 mg/mL LeIBP and the second group received the vitrification control ovaries. A group treated with 10 mg/mL LeIBP was the only one used for this experiment because it showed the best results in Experiment I. Mice were anesthetized by intraperitoneal injection of 30 mg/kg of Zoletil (Virbac, Carros, France) and 10 mg/kg of Rompun (Bayer, Leverkusen, Germany). Ovaries were then removed through bilateral incisions in the dorsal flank. All incisions were sutured with 4–0 silk suture silk within 10 min. The ovaries were then vitrified as described for Experiment I. Seven days after ovariectomy, vitrified ovaries were warmed as described above and were autotransplanted underneath the kidney capsules.

### Sample preparation

In Experiment I, ovaries were warmed and fixed immediately with 4% paraformaldehyde. In Experiment II, quick and humane sacrifice of mice was performed by cervical dislocation 7 days after transplantation to obtain the grafts and whole blood from each mouse. The collected ovaries were prepared for paraffin embedding. The blood serum was separated by centrifugation to perform an enzyme-linked immunosorbent assay (ELISA) to detect mouse follicle stimulating hormone (FSH).

### Morphological assessment and classification of ovarian follicles

Tissues were dehydrated, paraffin embedded and serially sectioned at 4-μm thickness, and the 5th section of tissue was mounted onto glass slides. The slides were stained with hematoxylin and eosin (Merck, Darmstadt, Germany) and then graded and analyzed for follicle counts. Each slide was read twice by a single experienced inspector (J. Lee). The averages of the follicle counts were used. Only follicles with a visible nucleus in the oocyte were counted to avoid double counting. Each follicle was classified according to the following categories [25]:

1. primordial: single layer of flattened pre-granulosa cells;
2. primary: single layer of granulosa cells, one or more of which was cuboidal;
3. secondary: two or more layers of cuboidal granulosa cells, with the antrum absent; or
4. antral: multiple layers of cuboidal granulosa cells with the antrum present.

The integrity of each follicle was evaluated using the following criteria [26]:

1. primordial/primary follicle: Grade 1 (G1), spherical with even distribution of the granulosa cells; Grade 2 (G2), granulosa cells pulled away from the edge of the follicle but with the oocytes still spherical; Grade 3 (G3), pyknotic nuclei, misshapen oocytes, or vacuolization;
2. secondary/antral follicle: G1, intact spherical follicle with evenly distributed granulosa and theca cells, small space, and spherical oocytes; G2, intact theca cells, disrupted granulosa cells, and spherical oocytes; G3, disruption and loss of granulosa and theca cells, pyknotic nuclei, and missing oocytes.

Atretic follicles were characterized by the presence of eosinophilia of the ooplasm, contraction and clumping of chromatin material, and wrinkling of the nuclear membranes in the oocytes.

### Assessment of apoptotic follicles

Apoptosis of follicles in the warmed and transplanted ovaries was evaluated as described previously [6]. Following deparaffinization and rehydration, each slide was treated with 0.8% proteinase K (Dako, Denmark) at room temperature for 15 min, incubated with a TUNEL reaction mixture (1:9 enzyme:label) for 1 h at 37°C in a humidified chamber in the dark, and then rinsed with D-PBS. The slides were then mounted in VECTASHIELD Mounting Medium with 4′,6-diamidino-2-phenylindole (DAPI) (Vector Laboratories, Burlingame, CA, USA), and examined under an inverted Zeiss AX10 microscope (Carl Zeiss, Oberkochen, Germany). Slides incubated without the TUNEL reaction mixture were used as negative controls and those incubated in 100 U/mL of DNase I were used as positive controls. Cells that were positive for the TUNEL assay exhibited green fluorescence. A follicle containing over 30% cells positive for green fluorescence was considered apoptotic.

### Immunohistochemical analyses

Following the TUNEL assay, immunohistochemical analyses were carried out to assess DSB and DDR using τH2AX and Rad51 antibodies, respectively [27]. Paraffin slides (4-μm thick) of the ovaries were baked, dewaxed, and then rehydrated in xylene, ethanol, and water. Rehydrated slides were microwave-heated for 20 min in an appropriate heat-induced epitope retrieval solution followed by 10 min with a peroxidase blocking solution (Dako) to inhibit endogenous peroxidase activity. The slides were then incubated with appropriate concentrations of τH2AX (1:100; Millipore, Billerica, MA, USA), Rad51 (1:100; Bioworld Technology, St. Louis, MO, USA), and CD31 (1:100; Abcam, Cambridge, UK) primary antibodies at room temperature for 1 h. Following incubation with τH2AX and Rad51 antibodies, the slides were incubated for 1 h at room temperature with an Alexa 594 conjugated anti-rabbit secondary antibody in blocking buffer (1:1,000; Invitrogen, Carlsbad, CA, USA). The slides were mounted with VECTASHIELD Mounting Medium containing DAPI (Vector Labs) and examined using a Zeiss AX10 fluorescence microscope (Carl Zeiss). Following incubation with the CD31 antibody, sections were treated with EnVision+ HRP (Dako, Carpinteria, CA, USA) for 30 min at room temperature and then treated with Liquid DAB+ Substrate (Dako, Denmark) for 10 min at room temperature. All sections were counterstained with hematoxylin (Dako, Denmark) and dehydrated in ethanol and xylene. Finally, the slides were mounted with Mounting Medium (Dako, Denmark) and examined under an inverted Zeiss AX10 microscope (Carl Zeiss). Follicles containing at least one nucleus stained with τH2AX were considered to contain DNA DSB. Follicles containing at least one nucleus stained with Rad51 were regarded as undergoing DNA DDR. In 2009, Brown and Holt demonstrated that the expression of Rad 51 was up-regulated by irradiation within 10 min [28]. Because the warming process in the present study took an additional 20 min, this was considered sufficient for DNA repair and for the expression of Rad51 to begin.

### Measurement of serum FSH levels

An ELISA kit (Endocrine Technologies, Newark, NJ, USA) was used to measure the concentration of serum FSH in the two different transplant groups (vitrification control and LeIBP-treated). According to the manufacturer’s instructions, FSH was measured by extrapolating the optical density reading of the ELISA plate at 450 nm, and concentrations were calculated using serial standard dilutions.

### Statistical analyses

The distribution of the follicle stages and normality in each sample were evaluated for each group. Data were analyzed by Student’s *t*-test, the Mann–Whitney test, the chi-square test, or ANOVA. Tukey’s test was used for post-hoc test. SPSS version 12.0 software (IBM, Chicago, IL, USA) was used for the statistical analyses, and a p value of < 0.05 was considered to indicate a statistically significant difference.

## Results

### Experiment I

#### Morphological evaluation of vitrified/warmed OT

**Fig 2:**
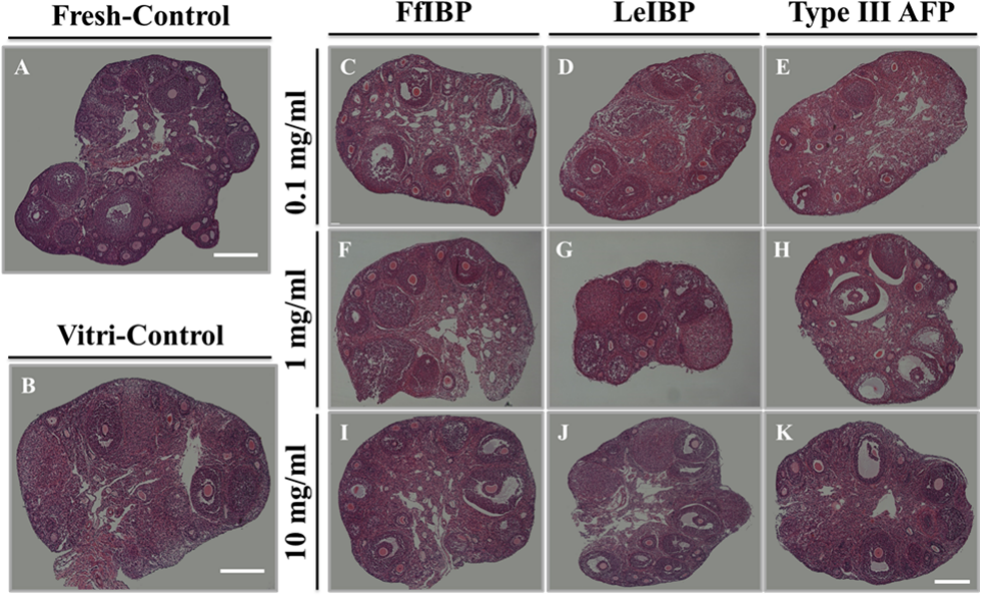
Representative images of hematoxylin and eosin stain for 11 groups according to type of antifreeze protein (AFP) and dose. (A) Fresh control (without cryopreservation); (B) vitrification control (cryopreservation without any AFP supplementation); (C–E) groups treated with 0.1 mg/mL AFP; (F–H) groups treated with 1.0 mg/mL AFP; (I–K) groups treated with 10 mg/mL AFP. (C, F, I) The FfIBP-treated group; (D, G, J) the LeIBP-treated group; and (E, H, K) the group treated with type III AFP. White bars indicate 500 μm; the magnification was 100×.

In total, 11 groups were examined morphologically and representative images are shown in Fig 2. The overall morphology of ovarian follicles in the fresh controls was superior to that seen in the other 10 groups. Most of the oocytes in follicles from fresh control OT were intact and clear, and the interstitial tissues appeared denser in the fresh control OT than in the other tissues (Fig 2A). As shown in Fig 2B, the vitrification/warming process may result in oocyte shrinkage and stromal damage. Similar to OT from the vitrification control group, oocytes and stromal cells in OT treated with 0.1 mg/mL or 1 mg/mL AFP exhibited cryodamage (Fig 2C–2H). However, OT treated with 10 mg/mL AFP (Fig 2I–2K) had well-preserved follicles, as compared with the vitrification control and OT treated with lower concentrations of AFP (Fig 2C–2H).

**Fig 3:**
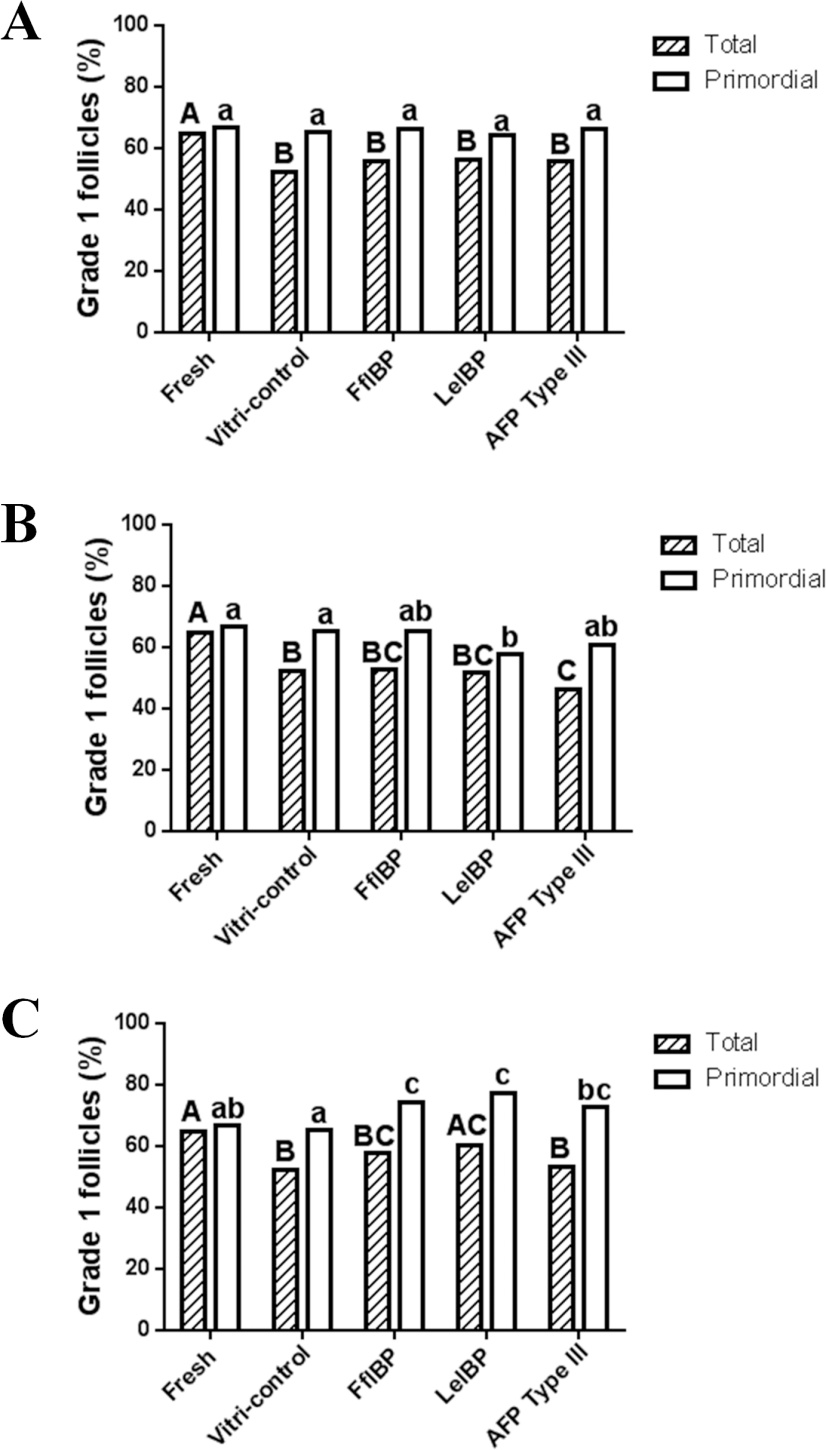
Percentages of total grade 1 follicles and primordial stage follicles in groups treated with (A) 0.1 mg/mL antifreeze protein (AFP), (B) 1.0 mg/mL AFP, and (C) 10 mg/mL AFP. Different of upper and lower case letters respectively indicate statistically significant differences among five groups in terms of total and primordial grade 1 follicle ratio, respectively (p<0.05).

In total, 5,790 ovarian follicles (fresh control: 754; vitrification control: 1003; FfIBP-treated: 1161; LeIBP-treated: 1333; and type III AFP-treated: 1489 follicles) were counted and classified by developmental stage and grade. The percentages of total and primordial G1 follicles was observed in the fresh and vitrification control OT, and OT treated with 0.1 or 1 mg/mL AFP (Fig 3A and 3B). In the vitrification control and AFP-treated OT, the total percentages of G1 follicles were significantly lower than that in the fresh control OT. However, the percentage of primordial G1 follicles in the fresh control OT was not different from that in all other groups, except for LeIBP-treated OT (Fig 3B; fresh control OT: 66.5%; vitrification control OT: 65%; FfIBP-treated OT: 65.3%; LeIBP-treated OT: 57.6%, and type III AFP-treated OT: 60.5%).

The total percentages of G1 and primordial G1 follicles was seen in the fresh control and vitrification control OTs, and the OTs treated with 10 mg/mL of the different AFPs (Fig 3C). Although the total percentages of G1 follicles in the vitrification control and most of the AFP-treated OTs were lower than those in the fresh control group, the percentage in OTs treated with 10 mg/mL LeIBP was comparable with that in the fresh control OT (fresh control OT: 64.6%; vitrification control OT: 52.2%, FfIBP-treated OT: 57.5%, LeIBP-treated OT: 60.2%; and type III AFP-treated OT: 53.4%). In addition, all AFP-treated OTs had a significant increase in the percentage of primordial G1 follicles as compared with the vitrification control OT. Moreover, the percentages of primordial G1 follicles were higher in FfIBP- and LeIBP-treated OTs than in fresh control OT (fresh control OT: 66.5%; vitrification control OT: 65%; FfIBP-treated OT: 74.2%; LeIBP-treated OT: 77.4%; and type III AFP-treated OT: 72.8%).

#### Analysis of apoptosis in vitrified/warmed OT

**Fig 4:**
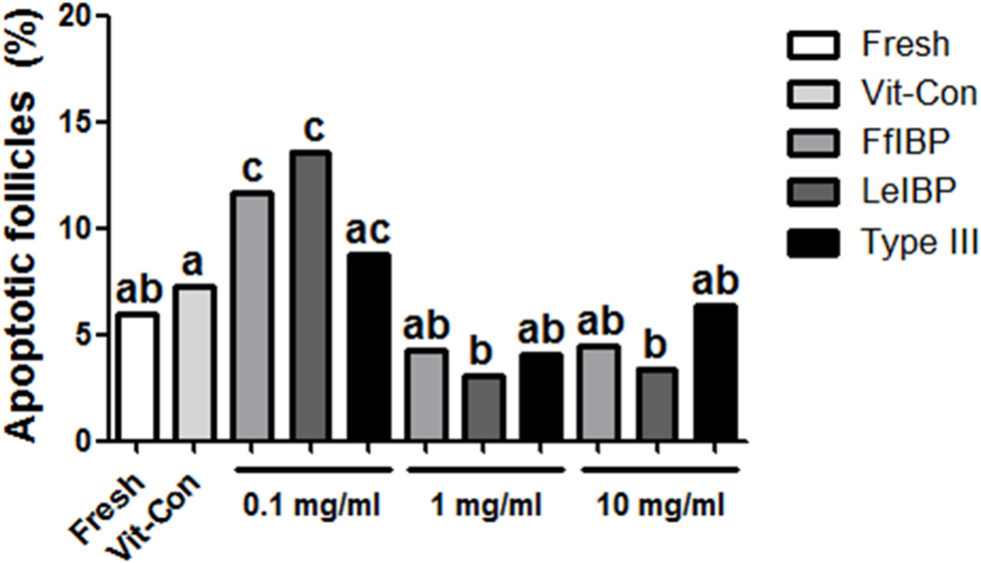
Percentage of apoptotic follicles in 11 groups after vitrification and warming procedures. Different letters indicate significant differences among five groups (p<0.05).

In OTs treated with 0.1 mg/mL of either FfIBP or LeIBP, the percentages of apoptotic follicles were greater than in the fresh and vitrification control OTs (Fig 4). However, OTs treated with 1 or 10 mg/mL AFP had percentages of apoptotic follicles that were similar to those seen in the vitrification control OT. Moreover, the percentage of apoptotic follicles in the LeIBP-treated OT was significantly less than that in the vitrification control OT.

#### Immunohistochemical analysis of vitrified/warmed OT

**Fig 5:**
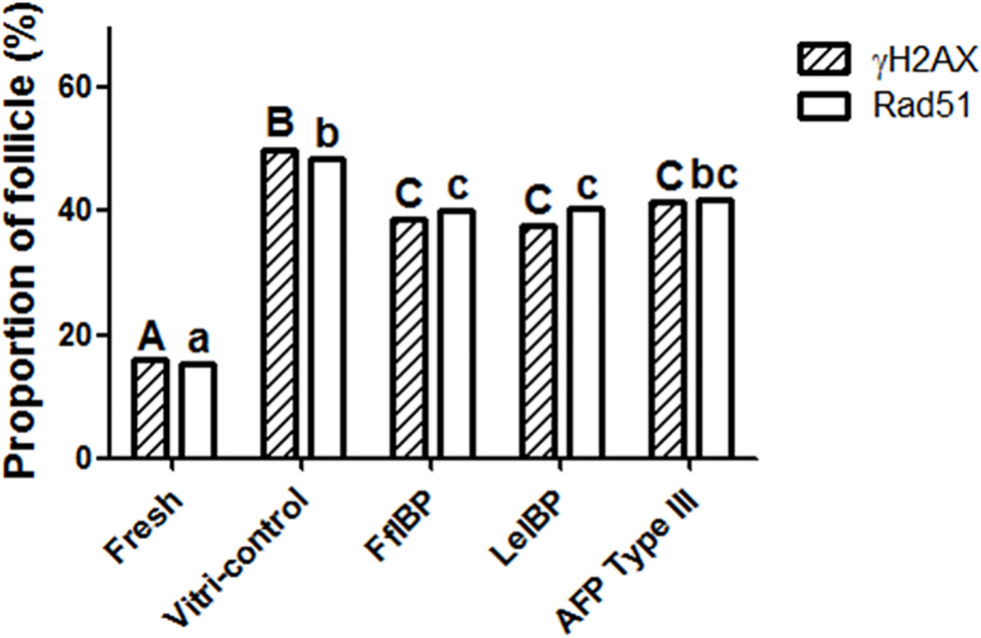
The percentage of τH2AX- and Rad51-positive follicles in the two control groups and the groups treated with 10 mg/mL of the different antifreeze proteins. Upper case and lower case letters respectively indicate significant differences among five groups in terms of τH2AX and Rad51, respectively (p<0.05).

As seen in Fig 5, the percentages of τH2AX positive follicles were significantly higher in the vitrification control and AFP-treated OTs than in the fresh control OT. AFP supplementation significantly decreased the percentage of τH2AX positive follicles, as compared with vitrification control OT (fresh control OT: 16%; vitrification control OT: 49.8%; FfIBP-treated OT: 38.6%; LeIBP-treated OT: 37.7%, and type III AFP-treated OT: 41.4%). The percentage of Rad51-positive follicles appeared to decrease with AFP treatment but was only statistically significant for FfIBP- and LeIBP-treated OTs compared with the vitrification control (fresh control OT: 15.2%; vitrification control OT: 48.6%; FfIBP-treated OT: 40.3%; LeIBP-treated OT: 40.5%; and type III AFP-treated OT: 41.9%).

### Experiment II

#### Histology, immunohistochemical analysis, and evaluation of apoptosis and serum FSH levels of recipient mice

**Fig 6:**
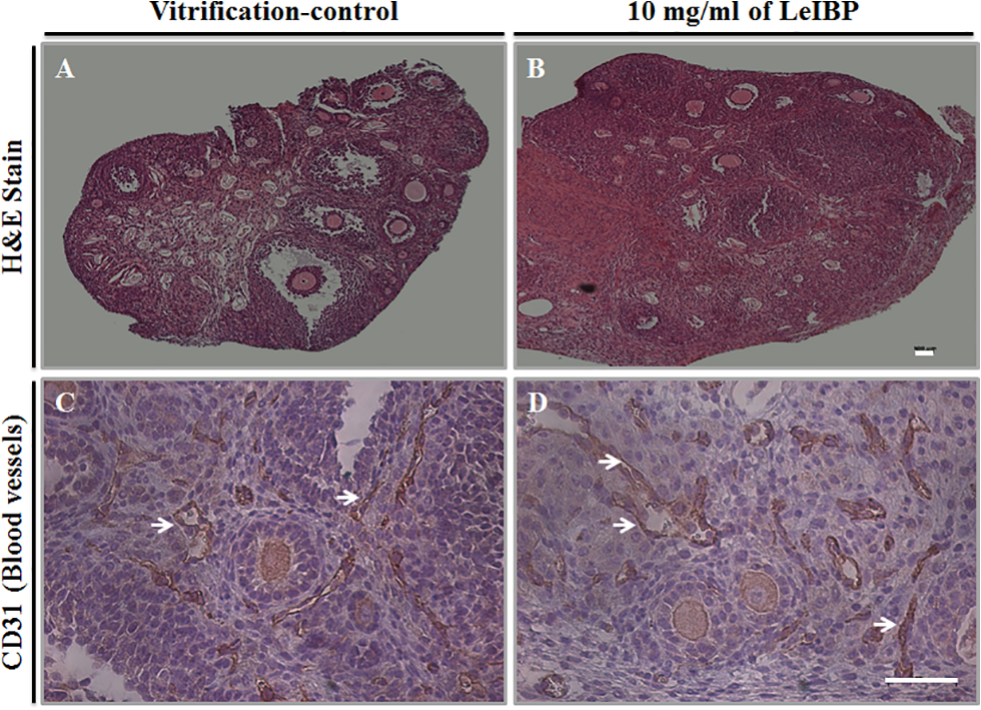
Histological assessment and immunohistochemical analysis for blood vessels (using the marker CD31) in transplanted ovarian tissue. Hematoxylin and eosin staining of grafts in (A) the vitrification-control group and (B) the LeIBP-treated group (100×). (C) CD31 expressed in endothelial cells in the vitrification-control group and (D) the LeIBP group (400×). Arrows indicate blood vessels, and white bars represent 100 μm.

**Fig 7:**
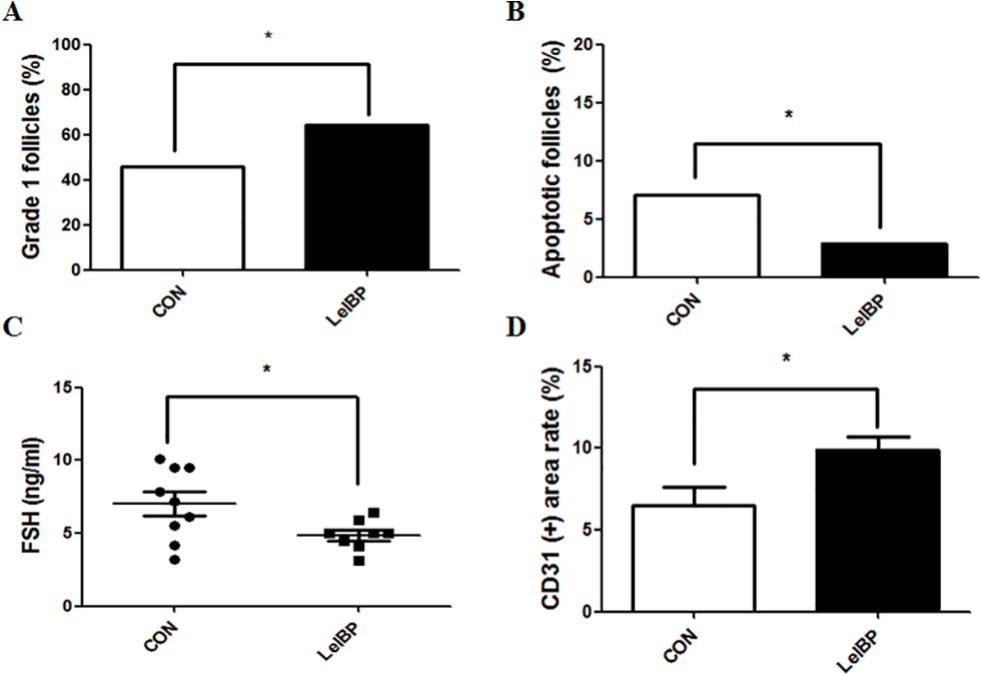
Comparisons of various parameters in the vitrification-control group and the LeIBP-treated group after ovarian tissue transplantation. (A) Percentage of total grade 1 follicles, (B) percentage of apoptotic follicles, (C) serum follicle stimulating hormone levels, and (D) CD31-positive area in both transplantation groups. Asterisks indicate significant differences compared with the vitrification-control.

The histological assessment and immunohistochemical was analyzed for CD31 after autotransplantation of both the vitrification control OT, and OT treated with 10 mg/mL LeIBP (Fig 6). There were many degraded follicles and damaged stromal cells evident in the vitrification control OT (Fig 6A). In contrast, there were lower numbers of degraded follicles in the LeIBP-treated than in the vitrification control OT (Fig 6B). The immunohistochemical detection of CD31 expression was carried out in endothelial cells in each graft (Fig 6C and 6D). Most of the CD31 expression was localized in late stage follicles and interstitial spaces. The percentages of G1 follicles and TUNEL-positive follicles was observed in the graft, the levels of serum FSH in the recipient mice on day 7, and the area of CD31-labeled endothelial cells in the grafts (Fig 7). LeIBP-treated OT had a statistically significant increase in the percentage of total G1 follicles. However, there were no significant differences in the percentages of primary, secondary, or antral G1 follicles between the control and the LeIBP-treated OTs (data not shown). For the LeIBP-treated grafts, the percentage of apoptotic follicles in OT was significantly lower, the serum FSH level in the recipient mice was significantly lower, and the area of CD31-positive cells in the graft was significantly greater than for the vitrification control grafts.

## Discussion

One of the main causes of cell death during cryopreservation is IR [29]. IR occurs constantly in nature due to moderate cooling and temperature fluctuations of frozen substances. During cryopreservation, IR during the thawing process causes cell membranes to rupture and causes cell dehydration, resulting in lethal damage to cells and tissues. It is believed that many freeze-tolerant organisms inhabiting cold environments have developed AFPs to ensure their survival [30]. Because AFPs are effective at inhibiting IR, they are beneficial for the cryopreservation of cells and tissues [20, 31–33]. The current study compared the cryoprotective efficacy of three types of recombinant AFPs in the vitrification of mouse OT. Addition of AFPs, especially 10 mg/mL of LeIBP, improved the OT density, follicle quality and decreased the apoptosis of follicles during vitrification-warming procedures. Furthermore, vitrified-warmed OTs with 10 mg/mL of LeIBP also increased the grade 1 follicle ratio and CD31-positive area in grafts as well as decreased apoptotic follicle ratio and serum FSH level after auto-transplantation of vitrified-warmed OTs.

Based on previous studies [11], 1 mg/mL of AFPs was used as the initial concentration. Concentrations that were 10 times lower and higher were also used. At both 0.1 and 1 mg/mL, no significant differences were observed between the AFPs in terms of follicular preservation at each of the developmental stages examined. However, at 10 mg/mL, AFPs provided cryoprotective effects in primordial follicle preservation, as compared with that observed in the vitrification control. This result indicates that, for the three types of AFPs tested, 10 mg/mL was sufficient to prevent cryodamage derived from the vitrification and warming processes.

In terms of primordial follicle preservation, the lowest grade 1 number was observed in the group supplemented with 1 mg/mL LeIBP. The reason why 1 mg/mL LeIBP decreased the percentage of primordial grade 1 follicles is unclear because we did not investigate the mechanism underlying the effects of LeIBP on OT vitrification. However, we are able to postulate some possible mechanisms based on other studies. Biphasic effects of AFPs were demonstrated by Wen and Laursen [34]. Moreover, other investigators have shown that various AFPs have different characteristics such as TH activity, ice-binding affinities, molecular weights, and structural features [11, 18, 19]. We assume that these differences, and biphasic effects derived from different types of AFPs, may result in the observed effects.

With respect to follicle apoptosis, a higher number of TUNEL-positive follicles was found in groups treated with 0.1 mg/mL FfIBP or LeIBP compared with that observed in fresh and vitrification-control OTs. In demonstrating the function of ‘type I antifreeze polypeptide (AFP type I)’, and its double-sided character in ice-growth inhibition [35], Wen and Laursen showed in 1992 that at a low concentration, AFP molecules bind randomly, and presumably reversibly, to the surface. In contrast, at high concentrations, intermolecular interactions occur and the surface becomes covered, either completely or in a patchwork pattern [36, 37]. Based on these previous studies, we suggest that the changeable actions (presumably binding randomly and reversibly to the surface) of AFPs at a low concentration could be the cause of the high number of apoptotic follicles in the groups treated with 0.1 mg/mL FfIBP or LeIBP. However, further studies are required to determine the exact mechanisms of various AFPs.

τH2AX is expressed at the site of DNA DSB, and Rad51 is localized at the DNA repair proteins [38]. Because high concentrations of three different AFPs reduced the percentage of apoptotic follicles, immunohistochemistry for τH2AX and Rad51 was performed to detect DNA DBS and DDR, respectively. The percentage of τH2AX-labeled follicles was significantly decreased in groups treated with the AFPs compared with that observed in the vitrification control. In addition, the percentage of follicles expressing Rad51 was significantly lower in FfIBP- and LeIBP-treated OTs compared with vitrification control OT. In 2005, Sak et al. demonstrated that there was a positive correlation between τH2AX and Rad51 expression, while Paull et al. reported that histone H2AX played a critical role in the recruitment of Rad51 to nuclear foci after DNA damage [39, 40]. Our data are consistent with the findings of these previous studies. According to these results, we assume that there is an increased activation of DNA DDR with increasing DNA DSBs. Therefore, cryodamage-induced Rad51 expression is positively correlated with τH2AX expression. These findings indicate that the anti-apoptotic effects of AFPs during cryopreservation are derived from preventing DNA DSBs.

In Experiment I, three AFPs with different properties were used. As shown in Table 1, FfIBP had the highest TH activity but the lowest IR inhibitory activity, while LeIBP showed the reverse. This result is consistent with that of a previous report by Yu et al. [41] who demonstrated that there was no obvious correlation between high TH activity and high IR inhibitory activity. In their IR inhibition experiment, moderately active type III AFP had higher (or comparable) IR inhibitory activity than did hyperactive AFPs. However, the mechanism by which AFPs inhibit IR and the reason why hyperactive AFPs do not show high IR inhibitory activity or vice versa, remained unclear. Our observations clearly showed that all AFPs were effective for cryopreserving OT and that the AFP with the highest IR inhibitory activity was the most beneficial. Lee et al. demonstrated that the ice-binding site in LeIBP was different from that in other AFPs and IBPs [19]. Although LeIBP has a conserved β-helical fold similar to that in canonical hyperactive AFPs, the ice-binding site is more complex and does not have a simple ice-binding motif.

In Experiment 2, the efficacy of 10 mg/mL LeIBP was analyzed 7 days after transplantation. This time was chosen because ischemia-reperfusion and hypoxia play major roles in follicle depletion in the first days after transplantation, and for about a week thereafter [4, 42–44]. Statistically significant improvements were observed in the percentage of G1 follicles, the percentage of apoptotic follicles, the serum FSH levels in recipient mice, and the extent of the CD31-positive area in each of the ovaries obtained from mice in the LeIBP-treated group, as compared with the vitrification control group. Shikanov et al. reported FSH concentrations in normal, ovariectomized, and transplant-recipient mice. Normal FSH levels ranged from 2 to 10 ng/mL, while in the absence of ovaries, the levels were 0 to 50 ng/mL, and decreased rapidly 7 days post-transplantation due to restored hormonal cyclicity [45]. Previous studies have suggested that an elevation of FSH indicates a reduction of ovarian reserve in the graft [46]. In our study, the serum FSH level was lower in mice transplanted with LeIBP-treated OT than in mice transplanted with the vitrification control OT, but remained within the normal range in both groups. Based on these results, we conclude that supplementation with LeIBP (10 mg/mL) during vitrification/warming procedures not only prevents cryoinjury to the grafts but also provides beneficial effects on ovarian function even after transplantation.

When Bagis et al. generated transgenic mice carrying a type III fish AFP gene, testicular and OT in the F3 generation were protected from damage during hypothermic storage [47]. In addition, these researchers evaluated the cryogenic effect of AFP type III on vitrified transgenic mouse OT, and the production of live offspring by orthotopic transplantation of cryopreserved mouse ovaries [3]. Their results indicated that the application of AFPs for cryobiology of mammalian reproductive cells and tissues is safe and stable, as they also showed that AFP genes were stably transcribed and expressed even in the seventh generation of transgenic mice [31]. Nonetheless, even though Bagis et al. provided a useful transgenic mouse model for investigating the biological functions of AFP in mammalian systems, more studies are required to assess the function of AFPs.

In the present study, we observed the restoration of function (hormonal assay) and used immunohistochemical and other analyses to verify the cryoprotective effects of three different AFPs. Nevertheless, our study has some limitations. We did not investigate the exact mechanism underlying the beneficial effects derived from AFPs during vitrification-warming procedures in the transplantation process. Moreover, we did not evaluate the impact of AFPs on oocyte quality and embryonic development. Finally, there was also no slow freezing control in this study, somewhat limiting our conclusions.

## Conclusions

This is the first study to compare the cryoprotective effects of different AFPs in OT cryopreservation and transplantation. Our study demonstrated the cryoprotective effects of AFPs, especially a high concentration of LeIBP, on OT during the vitrification/warming process. The findings of this study provide a foundation for further research on the effects and mechanisms of AFPs in human OT. To date, no study has investigated AFPs in humans. Therefore, further research is required to investigate the effects and mechanisms of AFPs in human OT.

## Supporting Information

S1 File Supplementary minimal raw data set to replicate experimentation. (ZIP)
